# P-2048. COVID-19 XBB.1.5 Vaccine Uptake Based on State Vaccine Registries Compared to National Survey Data

**DOI:** 10.1093/ofid/ofae631.2204

**Published:** 2025-01-29

**Authors:** Angela Cook, Kathleen M Andersen, Matthew A Brouillette, Leah McGrath, John M McLaughlin

**Affiliations:** Pfizer, Brooklyn, New York; Pfizer Inc., New York, New York; Pfizer Inc., New York, New York; Pfizer, Brooklyn, New York; Pfizer, Brooklyn, New York

## Abstract

**Background:**

Prior to May 11, 2023, all COVID-19 vaccinations were required to be reported by states to the Centers for Disease Control and Prevention. In the post-pandemic era, national vaccination uptake is now estimated using the National Immunization Survey (NIS). While survey methodology attempts to achieve random sampling of the population and accurate assessment of vaccine use through self-report, it is unknown how these estimates compare to state vaccine registries. We aimed to compare estimated COVID-19 vaccine uptake during the 2023-2024 season from state vaccine registries with estimates from the NIS.

**Figure 1.**
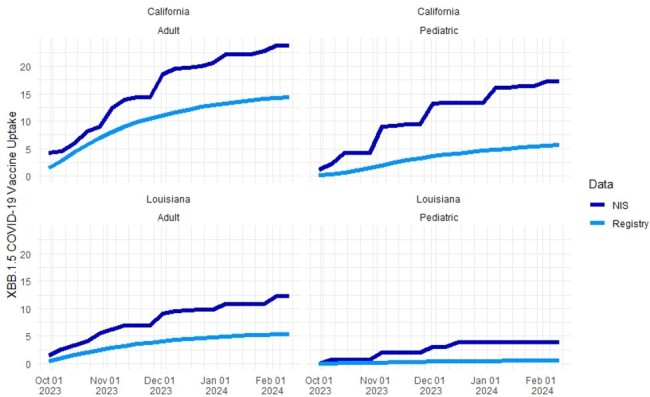
Uptake of XBB.1.5 COVID-19 vaccine in state vaccine registries versus national survey estimates, September 2023 - February 2024

**Methods:**

We utilized two COVID-19 state vaccination registries where COVID-19 vaccine reporting was required: CAIR2 in California and LINKS in Louisiana. We identified COVID-19 XBB.1.5-adapted vaccines administered between September 11, 2023, and February 10, 2024, using CVX vaccine codes 308 - 313. California and Louisiana 2023 population estimates were obtained from the U.S. Census Bureau and used as the denominator to calculate population-level uptake. For the same time period, state-specific COVID-19 vaccine uptake estimates were also obtained from state-specific NIS data for California and Louisiana as a comparison.

**Results:**

At the end of the study period, 14.4% of adults and 5.6% of children in California were documented as vaccinated in the CAIR2 registry compared to NIS estimates that 23.8% (1.6x) of adults and 17.3% (3.1x) of children in California had been XBB.1.5 vaccinated (Figure). For Louisiana, 5.4% of adults and 0.6% of children were found in LINKS registry data compared to NIS estimates that 12.3% (2.3x) of adults and 3.9% (6.5x) of children were XBB.1.5 vaccinated.

**Conclusion:**

XBB vaccination coverage was low overall but was substantially lower when measured using state vaccine registry data compared with national survey data, especially for children. Efforts to improve COVID-19 vaccination are warranted, as are future studies to determine why COVID-19 uptake appears higher in national survey data compared to what is captured in state registries.

**Disclosures:**

Angela Cook, MS, Pfizer: Employee|Pfizer: Stocks/Bonds (Public Company) Kathleen M. Andersen, PhD MSc, Pfizer Inc.: Employee|Pfizer Inc.: Stocks/Bonds (Public Company) Matthew A. Brouillette, MPH, Pfizer Inc.: Employee|Pfizer Inc.: Stocks/Bonds (Public Company) Leah McGrath, PhD, Pfizer Inc.: Employee|Pfizer Inc.: Stocks/Bonds (Public Company) John M. McLaughlin, PhD, Pfizer: Employee|Pfizer: Stocks/Bonds (Public Company)

